# Treatment of Disease in Children

**Published:** 1887-12

**Authors:** 


*Treatment of Disease in Children.* By ANGEL MONEY, M.D. Pp. 560. London: H. K. LEWIS. 1887.

There is not much scope for originality in the production of a book of Disease in Children. For, firstly, many books have already been written upon the same subject; and secondly, diseases in children are after all not very different from diseases in adolescents and adults. Dr. Money has done well with his subject, far better than most. In his preface he says somewhat grandly that he has endeavoured to "present," from his accumulated material, "a co-ordinate microcosm for the inco-ordinate chaos." This microcosm, it may be noted, runs on to 560 pages. "It is rare," he says, "that in treatment our indiscretion serveth us better than our judgment." "In medicine, as in the universe, all things are relative," he says in the introduction. Probably it is because we so seldom meet with this mood of philosophy in medical books nowadays that we are so much struck with it when we do meet it. But it is not all philosophic generalising; on the contrary, there is a great deal of personal, dogmatic, and exceedingly concrete statement. "All through the book he has," as he tells us, "emphasised that treatment he is accustomed to adopt." There is rarely any doubt as to the weight of this emphasis.

The book is full—too full indeed. The truth of the matter is that most books on children's diseases are simply books on general medicine boiled down where the diseases are common to the whole race, and stretched out where they are peculiar to children. They are created by a man who happens to be connected with a children's hospital, and for people connected with that hospital. They are of little use to the general physician or the general practitioner. For instance, Dr. Money gives an account of "Hydatid of Liver" in his book. Now is this disease in any sense of the word peculiar to children, or even common in them? And if anyone wanted to know about Hydatid of Liver, would he ever dream of going to a book on children's diseases for it? We say nothing as to the manner in which the disease is handled; we simply doubt the propriety of its being handled here at all. And what we say of hydatids is applicable to many other diseases. Still, Dr. Money's work is, in our opinion, quite one of the best of its sort.

